# The Real-Time and Patient-Specific Prediction for Duration and Recovery Profile of Cisatracurium Based on Deep Learning Models

**DOI:** 10.3389/fphar.2021.831149

**Published:** 2022-02-04

**Authors:** Kan Wang, Binyu Gao, Heqi Liu, Hui Chen, Honglei Liu

**Affiliations:** ^1^ Department of Anesthesiology, China-Japan Friendship Hospital, Beijing, China; ^2^ School of Biomedical Engineering, Capital Medical University, Beijing, China; ^3^ Beijing Key Laboratory of Fundamental Research on Biomechanics in Clinical Application, Capital Medical University, Beijing, China

**Keywords:** deep learning–artificial neural network, Long Short-Term Memory, GRU (gated recurrent unit), temporal sequence analysis, general anesthesia

## Abstract

During general anesthesia, how to judge the patient’s muscle relaxation state has always been one of the most significant issues for anesthesiologists. Train-of-four ratio (TOFR) monitoring is a standard method, which can only obtain static data to judge the current situation of muscle relaxation. Cisatracurium is a nondepolarizing benzylisoquinoline muscle relaxant. Real-time prediction of TOFR could help anesthesiologists to evaluate the duration and recovery profile of cisatracurium. TOFR of cisatracurium could be regarded as temporal sequence data, which could be processed and predicted using RNN based deep learning methods. In this work, we performed RNN, GRU, and LSTM models for TOFR prediction. We used transfer learning based on patient similarity derived from BMI and age to achieve real-time and patient-specific prediction. The GRU model achieved the best performance. In transfer learning, the model chosen based on patient similarity has significantly outperformed the model chosen randomly. Our work verified the feasibility of real-time prediction for TOFR of cisatracurium, which had practical significance in general anesthesia. Meanwhile, using the patient demographic data in transfer learning, our work could also achieve the patient-specific prediction, having theoretical value for the clinical research of precision medicine.

## Introduction

It has always been one of the most important issues for anesthesiologists to accurately determine the state of muscle relaxation of patients when neuromuscular blocking agents are used during general anesthesia. Monitoring of the degree of neuromuscular block should be part of standard perioperative monitoring (Kopman, 2010). As a common muscle relaxant, cisatracurium has been widely used because of its advantages including a short metabolic cycle, no metabolism by the kidney, and less release of histamine. TOFR (Train-of-four ratio) is a primarily used method that clinicians use to monitor muscle relaxation during surgery in patients under general anesthesia (Naguib et al., 2018). However, current muscle relaxation monitoring methods can only obtain static data to judge the real-time status of muscle relaxation (Bergeron et al., 2001; Dong and Li, 2012). Therefore, there is an urgent need for TOFR prediction in order to predict the state of muscle relaxation in advance and manage it accordingly.

Machining learning algorithms are applied to build classifier through integrating heterogeneous and complex clinical data, which could provide insights into clinical decision making (Liu et al., 2021a; Mikles et al., 2021). Recently, deep learning methods have profoundly impacted clinical research because of their simplicity, efficient processing and state-of-the-art performance (Liu et al., 2021b; Chen et al., 2021). Of different kinds of deep learning models, recurrent neural networks (RNN) have a recurrent structure that allows parameters to be dependent on adjacent parameters, having an advantage in representing ordered structure in data. Gated recurrent units (GRUs) and long short-term memory (LSTM) are two popular kinds of RNN mechanisms. These RNN-based models have been efficiently performed on temporal data, such as the prediction/forecasting of COVID-19 cases number (Shyam Sunder Reddy et al., 2020), ambulatory sleep detection (Sano et al., 2019), blood pressure estimation (Li et al., 2020), and so on.

During general anesthesia, the TOFR stimulation was repeated every 15 s. Therefore, the TOFR data could be recognized as temporal sequence data. Traditional methods for pharmacodynamics of neuromuscular blockade prediction concluded regression and pharmacokinetic model. Temporal sequence values could be processed and predicted using RNN based deep learning methods. However, as the best known of our knowledge, there are no previous studies on drug-related research. Furthermore, in the previous traditional time-series prediction, the model was trained by the earlier time-series data and to predict the following data (Shyam Sunder Reddy et al., 2020). Since the model was not already constructed, these models couldn’t obtain the real-time prediction under general anesthesia, which lasted only about several hours or less and was on the order of seconds. To complete the real-time prediction of TOFR, we designed the transfer model based on patient similarity. Before general anesthesia, we chose the most appropriate deep learning model from the existing training models based on the similarity between the patient in the test sets and the patients in the training sets. Since the model construction was not replied on the front part of the TOFR sequence, the method could achieve the real-time prediction. We used BMI and age to estimate the similarities between patients. Previous studies had discussed the relationship between age and BMI with cisatracurium or muscle relaxants (Kitts et al., 1990; Sorooshian et al., 1996; Pühringer et al., 1999; Pühringer et al., 2002; Leykin et al., 2004; Arain et al., 2005; Xiaobo et al., 2012). These studies could verify the rationality of patient similarity measurement in terms of age and BMI.

In this study, we applied the RNN based deep learning methods to predict the real-time TOFR of cisatracurium by integrating temporal sequence data and static factors. Transfer learning was performed according to the patient similarity to get accurate and reliable patient-specific predictions. Our model could facilitate the application of deep learning methods into clinical decision support.

## Methods

**FIGURE 1:**
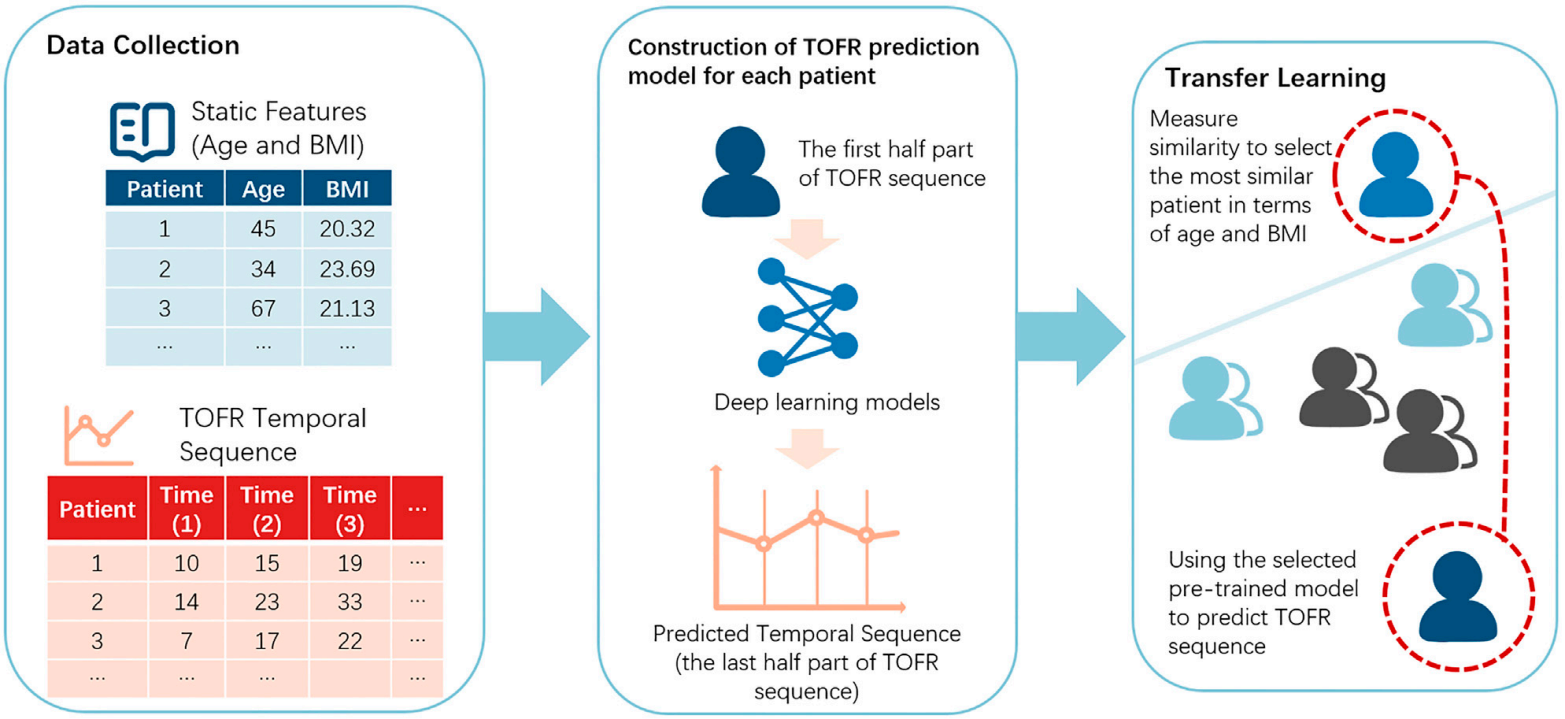
The workflow of this research.

The proposed workflow for prediction of TOFR data prediction was shown in Figure 1. We collected the static features (age and BMI) and TOFR temporal sequence for each patient. In the prediction step, we build the deep learning models to predict the TOFR sequence for each patient. When faced with a new patient, we used the transfer learning method by selecting the most similar patient, and use its pre-trained model to predict the TOFR sequence of the new patient.

### Ethics

This clinical trial study of cisatracurium (no. 2018-96-K70) was approved by the China-Japan Friendship Hospital and registered at a publicly accessible clinical trial registration site (Chinese Clinical Trial Registry, ChiCTR1800020180).

### Inclusion and Exclusion

The study staff recruited the participants between October 2018 and October 2019 at the China-Japan Friendship Hospital. Entrants gave written, informed consent to participate. Inclusion criteria were an age between 18 and 75 years, a body mass index<35 kg/m^2^, an American Society of Anesthesiologists physical status I to II, and a scheduled elective arthroscopic surgery or total knee arthroplasty with an expected duration of at least 60 min necessitating muscle relaxant administration for intubation of the trachea; full relaxation for the surgery was not required. Exclusion criteria were bronchial asthma, chronic obstructive pulmonary disease, neuromuscular disease, suspected malignant hyperthermia, significant hepatic or renal dysfunction, glaucoma, allergy to the drugs used in this study, and the use of medication known to alter the effect of neuromuscular blocking agents. In addition, patients who participated in another study within 30 days were excluded, nor were pregnant or breastfeeding women.

### Anesthesia Procedures

In the operating room, an IV cannula was inserted in a forearm vein, and vital sign monitoring was started. Routine monitoring, such as electrocardiography, noninvasive blood pressure monitoring, pulse oximetry, and BIS monitoring (BIS VISTA; Medtronic, Ireland), was applied before anesthesia induction. Patients were given 0.02 mg/kg midazolam 10 min before the induction of anesthesia. The patients then received prophylactic antibiotics depending on the type of surgery. Anesthesia was induced with IV propofol (1.5–2.0 mg/kg), cisatracurium (0.2 mg/kg) and fentanyl (2.0 μg/kg) and maintained with remifentanil (0.1–0.3 μg·kg^−1^·h^−1^) and propofol (50–100 μg·kg^−1^·h^−1^). The propofol target was titrated to keep the BIS between 40 and 60, and the remifentanil target was adjusted in response to systemic blood pressure during anesthesia maintenance. The patients’ lungs were manually ventilated with oxygen using a facemask until intubation of the trachea. Normocapnia was ensured, the oxygen saturation was maintained above 96%, and the esophageal temperature was maintained above 36°C.

### Neuromuscular Monitoring

Neuromuscular monitoring was carried out using TOF-Watch SX acceleromyography (Organon Teknika B.V., Netherlands). We recorded the adductor pollicis muscle contractions in response to ulnar nerve stimulation. The piezoelectric probe of the acceleromyograph was attached to the tip of the thumb. A hand adapter ensured the preload of the thumb while ensuring that it continued to return to its original position. The forearm and the fingers were immobilized, and surface skin electrodes were placed over the ulnar nerve proximal to the wrist. A TOFR mode of stimulation was started and repeated every 15 s for 3 min followed by a 5-s tetanic train of 50 Hz to stabilize the signal. Two min later, automatic calibration was carried out (the calibration to set supramaximal current intensity and calibrate the device). TOFR stimulation was recommenced by delivering supramaximal square wave stimuli with a duration of 0.2 ms and a frequency of 2 Hz until the signal was stable. If the signal was not stable, the calibration was repeated.

Data were recorded and stored on a computer by a recorder. Once the neuromuscular recording was stable, 0.20 mg/kg cisatracurium (4 times ED95) was injected IV, and the trachea was intubated when the muscle response to TOF stimulation disappeared. If surgical relaxation was necessary, 0.05 mg/kg cisatracurium was administered. The TOFR stimulation was automatically delivered at every 15 s interval.

### Deep Learning Methods- RNN, GRU and LSTM

The TOFR value of each patient could be regarded as a temporal sequence. RNN-based deep learning methods are widely used in sequence data processing. RNN has a recurrent structure which allows its parameters to be dependent on adjacent parameters. GRU is a gating mechanism in RNN, introduced in 2014 by Kyunghyun Cho et al. The GRU is like an LSTM with a forget gate but has fewer parameters, as it lacks an output gate (Chung et al., 2014). RNN model suffers from short-term memory. If a sequence is long enough, it will be hard to carry information from earlier time steps to the following ones.

In previous studies, GRU was verified to have better performance on certain less frequent and smaller datasets (Gruber and Jockisch, 2020). Therefore, we used GRU for the TOFR temporal sequence of all 83 patients. We applied LSTM and RNN as the baseline models for comparison with GRU. The TOFR temporal sequence data could be presented as X = {*x*_*1*_ *, x*_*2*_ *… x*_*n*_}. We divided the whole sequence data into training and test parts, and the test part was estimated. Linear interpolation was used to fill the missing data in the train part. During the prediction, we divided the temporal sequence into multiple input/output patterns, where four time steps were regarded as input and one time step was used as output for the one-step prediction.

We created the deep learning neural network models in Python using Keras. Root mean square error (RMSE) was used to evaluate the performance of models, as shown in equation (Kopman, 2010), where n is the number of time points. *x* and $\hat{x}$ represented the observed TOFR and the predicted TOFR, respectively.

$$\sqrt{\frac{1}{n}\sum_{i=1}^{n}{\left(x_{i}-\overset{\wedge }{x_{i}}\right)}^{2}}$$ (1)

### Transfer Learning Based on the Patient Similarity

Transfer learning is a machine learning technique where a model trained on one problem is re-purposed on a different but related problem (Zhuang et al., 2021). Two common approaches to transfer learning are as follows: develop model approach and pre-trained model approach. In the pre-trained model approach, a source model is chosen from all available models and then be used as the starting point for a model on the second task of interest.

In this work, while the patient is under general anesthesia, it is not practical to construct a completely new model based on the front part of the TOFR data. Therefore, to achieve the patient-specific and real-time prediction of TOFR temporal sequence data, we chose a pre-trained model from the available constructed models according to the demographic information. Therefore, no parameters in the model needed to be computed, which could be directly transferred from the chosen model.

We built the corresponding deep learning models for each patient in the training set, obtaining their relevant parameters.

Then, we measured the patient similarity between patients in the test set and patients in the training set according to age and BMI. For each patient in the test set, we search for the patients within a 2-year difference. Of these patients, we then find the one with the minimum difference of BMI. We defined the selected patient chosen as the most similar patient and then decided its model to be the transfer learning model. Thus, no more new model needed to be constructed while the patient was under general anesthesia.

## Results

### Dataset

Between October 2018 and October 2019, patients undergoing routine elective surgery were assessed for eligibility. Patients were excluded if the TOFR was less than 90 at the end of the surgery. Finally, 83 patients were enrolled in this study. For the TOFR temporal sequence X = {*x*_*1*_ *, x*_*2*_ *… x*_*n*_}, n ranged from 119 to 228. Every minute had four time points.

### TOFR Temporal Sequence Prediction Using Deep Learning Methods

**FIGURE 2:**
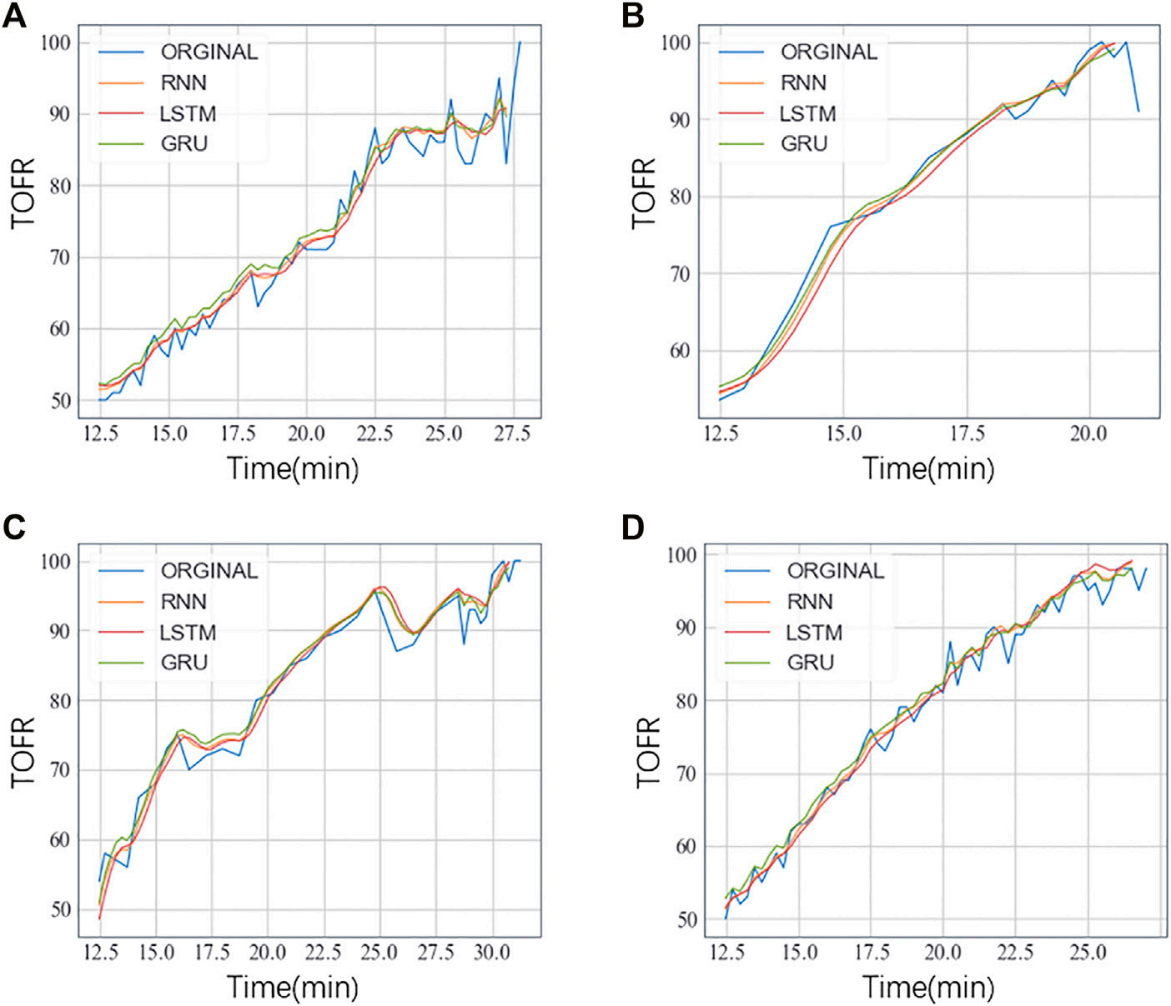
The TOFR curves of four patients **(A-D)** randomly selected.

We build the deep learning model GRU to predict the TOFR sequence for each patient. We also applied LSTM and RNN as the baseline models. For each sequence X = {*x*_*1*_ *, x*_*2*_ *… x*_*n*_}, the first half of X were training data and the second half of X were the testing data. The mean RMSE was computed and presented in Table 1. We randomly chose four patients and showed the TOFR curves in Figure 2. All models could get good performance. The result of GRU outperformed other two models.

**TABLE 1:** Result of TOFR temporal sequence prediction. Mean value ± std (standard deviation) was presented.

	RNN	GRU	LSTM
mean RMSE	3.37 ± 1.37	2.53 ± 0.86	4.14 ± 2.30

### TOFR Temporal Sequence Prediction Based on Transfer Learning

To verify we could use the similarity in terms of BMI and age in transfer learning, we divided all pairwise patients into two group: patients with similarity (within 2-year difference and 3 kg/m^2^ difference), patients without similarity. For each pairwise patients, TOFR sequences was predicted using the pre-trained model of the other patient. We computed the RMSE and presented the mean RMSE after the prediction of all pairwise TOFR sequences in Table 2. The two groups had a significant difference (*p* < 0.05, T test).

**TABLE 2:** The mean RMSE between all pairwise patients. Mean value ± std (standard deviation).

	Mean RMSE (patients with similarity)	Mean RMSE (patients without similarity)	*p* Value
GRU	2.75 ± 0.80	3.05 ± 1.09	0.048

**FIGURE 3:**
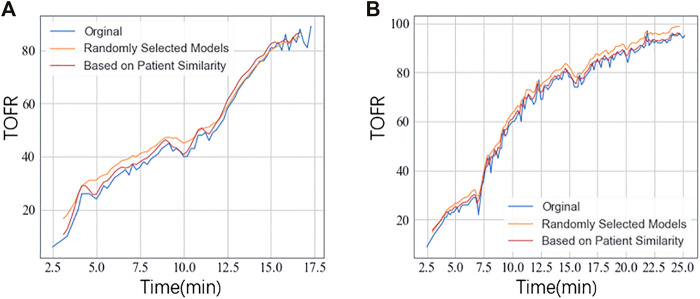
The TOFR curves of two patients **(A,B)** based on transfer learning. GRU model was performed.

We used the leave-one-out cross-validation to evaluate the performance of prediction models. We build the deep learning models for each patient in the training set, as described in *TOFR Temporal Sequence Prediction Using Deep Learning Methods*. For each patient, based on transfer learning, a model in the training set was chosen based on patient similarity and performed. In contrast, we randomly selected a model in the training set and compared its prediction result with the model selected based on patient similarity. We presented the mean RMSE in Table 3. We also showed the TOFR curves of prediction results from two patients using GRU (Figure 3), RNN (Supplementary Figure S1), and LSTM (Supplementary Figure S2).

**TABLE 3:** Results of TOFR prediction based on transfer learning using leave-one-out method. Mean value ± std (standard deviation).

	Mean RMSE (patient similarity)	Mean RMSE (randomly selected model)
RNN	4.27 ± 2.23	4.91 ± 2.42
GRU	2.75 ± 0.80	3.05 ± 1.09
LSTM	3.89 ± 1.58	4.09 ± 1.60

From the RMSE results and TOFR curve, for all the deep learning models, the model derived based on patient similarity was much better than the model randomly chosen. In addition, GRU obtained the best performance than the other two models. The prediction performance of LSTM was better than RNN.

## Discussion

Cisatracurium is a widely used neuromuscular blocking agent. In practice, on the one hand, it is necessary to meet the requirements of surgical muscle relaxants; on the other hand, it is essential to reduce the incidence of postoperative residual neuromuscular block. Therefore, the cisatracurium or antagonist should be given at the right time. TOFR is a standard method to monitor muscle relaxation. In this study, we designed the deep learning framework to realize the real-time and patient-specific prediction of TOFR. Thus we could predict and analyze the duration and recovery profile of cisatracurium according to TOFR values.

We used transfer learning to achieve the real-time prediction of TOFR. In addition, age and BMI were used to measure the patient similarity between patients in the test set and training set. Some studies had discussed the relationship between these factors with cisatracurium or muscle relaxants, providing the theoretical basis of patient similarity measurement. With increasing age, the body’s water capacity and muscle strength gradually decreases, the body fat content gradually increases, and a decrease in cardiac output leads to the decline of liver and kidney blood flow. These factors will affect the muscle response to muscle relaxants (Pühringer et al., 2002; Arain et al., 2005). Therefore, although the plasma clearance of cisatracurium did not differ between young and older patients (Sorooshian et al., 1996), the duration of action was not necessarily the same. Besides, with 77% of the cisatracurium in the body metabolized through Hofmann degradation, Kitts and colleagues suggested a significant difference in pharmacokinetics between elderly and young people, resulting in a slightly prolonged nerve bundle block in elderly patients (Kitts et al., 1990). As age increased, renal function gradually decreased, which also had a particular effect on pharmacodynamics (Xiaobo et al., 2012). There are also many contradictory conclusions about the impact of BMI on muscle relaxants (Pühringer et al., 1999; Xiaobo et al., 2012). Pühringer et al. (1999) demonstrated that the metabolic rate of vecuronium in obese patients was not different from that of normal-weight patients. On the other hand, Leykin et al. (2004) found that the metabolic time of cisatracurium was prolonged in morbidly obese patients. According to Yigal’s result, morbidly obese patients had better choose lean body weight to calculate the dosage of muscle relaxants.

In this work, TOFR values for each patient during general anesthesia were regarded as temporal sequences. RNN-based deep learning models have been widely used in the processing and prediction of temporal sequence. GRU exhibited better performance than other RNN-based models both with and without transfer learning. The GRU is like an LSTM mechanism with a forget gate but has fewer parameters and lacks an output gate. In previous studies, GRU had better performance on smaller datasets (Gruber and Jockisch, 2020). In this work, TOFR temporal sequence data could be seen as small dataset since the TOFR of each patient contained only less than one hundred values. For the other two methods, LSTM obtained a better prediction result than RNN. In the processing of temporal sequence, there can be lags of unknown duration between values in sequence. Therefore, compared with RNN, LSTM models are more suited to processing and making predictions based on temporal data.

Transfer learning obtains rapid progress or improved performance when modeling different but related problems. It is a popular approach in deep learning tasks, such as computer vision and natural language processing. In this work, to achieve the real-time TOFR temporal sequence prediction, a pre-trained source model was chosen from the available constructed model and then be used as the starting point. Demographic information was used to calculate the patient similarity, which was the criterion for model selection. Therefore, our study could also realize the patient-specific prediction of the TOFR temporal sequence. Furthermore, demographic information was easy to capture before the general anesthesia. Compared with models chosen randomly, pre-trained models chosen based on patient similarity had much better performance in prediction, which further verified the meaning of clinical decision making according to precision medicine.

## Conclusion

In this work, we introduced a deep learning framework to predict TOFR temporary sequence; further, we can obtain the duration and recovery profile of cisatracurium by predicting the TOFR temporal sequence. We proved the feasibility of the RNN-based deep learning models for the real-time prediction of TOFR temporal sequence. With the patient similarity, we can obtain patient-specific prediction. Our research is of crucial theoretical value and practical significance in the clinical application of muscle relaxants.

## Data Availability

The data analyzed in this study is subject to the following licenses/restrictions: human participants. Requests to access these datasets should be directed to the corresponding authors.
