# Supplementary material for: The Real-Time and Patient-Specific Prediction for Duration and Recovery Profile of Cisatracurium Based on Deep Learning Models
Source: Front Pharmacol. 2022 Feb 4;12:831149. doi: 10.3389/fphar.2021.831149 (PMC8854501; doi:10.3389/fphar.2021.831149)
Supplement: Supplementary file 1 [file Presentation1.pdf]

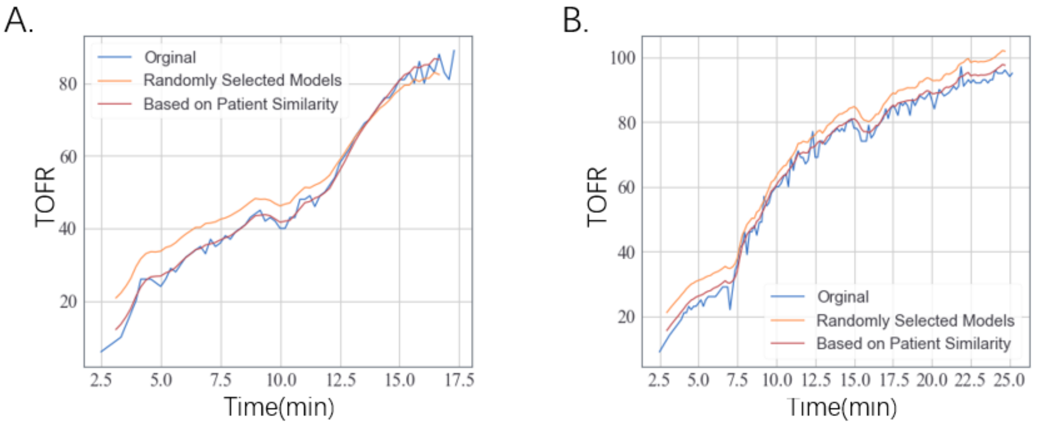

Supplementary Figure 1. The TOFR curves of two patients based on transfer learning. RNN model was performed.

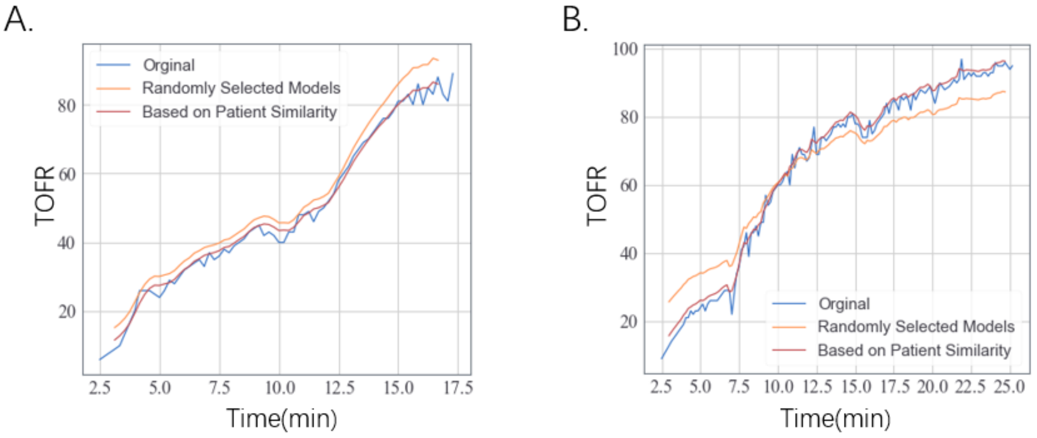

Supplementary Figure 2. The TOFR curves of two patients based on transfer learning. LSTM model was performed
